# Effect of Adherence to Smartphone App Use on the Long-term Effectiveness of Weight Loss in Developing and OECD Countries: Retrospective Cohort Study

**DOI:** 10.2196/13496

**Published:** 2021-07-12

**Authors:** Myeunghee Han, Sang Youl Rhee

**Affiliations:** 1 Daegu Health High School Daegu Republic of Korea; 2 Department of Endocrinology and Metabolism Kyung Hee University School of Medicine Seoul Republic of Korea

**Keywords:** low-income countries, Organization for Economic Co-operation and Development, body weight, mobile app self-management, diet, exercise, mobile phone

## Abstract

**Background:**

Globally, 71% of deaths occur due to noncommunicable diseases (NCDs). Poor diet quality and physical activity have a significant impact on NCDs. At present, behavior change interventions using smartphone apps have rapidly increased worldwide to prevent NCDs. However, most previous studies on the use and effectiveness of apps have been conducted in Organization for Economic Co-operation and Development (OECD) countries. As such, relevant research in low-income countries is scarce.

**Objective:**

This retrospective cohort study aims to investigate the characteristics of adherence to the use of the Noom app. We also aim to compare the effects of using the app on body weight changes over time according to adherence to the use of the app between users in low-income and OECD countries. In addition, the differences in weight loss are compared among users who use the free and paid versions of the app.

**Methods:**

A secondary data analysis was conducted using repeated measures. The data were collected from users in low-income countries (n=312) and OECD countries (n=8041) who used the app for 12 months. The app provided programs for the self-monitoring of physical activity, dietary intake, and body weight. Descriptive statistics, independent two-tailed *t* tests, chi-square tests, and linear mixed models were used for the analysis.

**Results:**

During the first 3 months of using the Noom app, users from OECD countries entered data into the app more frequently; however, users in low-income countries entered data more frequently from 3 months to 12 months. Users in OECD countries consumed significantly more calories than those in low-income countries for 12 months. The body weight of all users significantly decreased over time (−1.8 kg; *P*<.001); however, no statistically significant differences in the change in body weight for 12 months were observed between users from low-income and OECD countries (β=−.2; *P*=.19). The users who frequently monitored their lunch (β=−.1; *P*<.001), dinner (β=−.1; *P*<.001), body weight (β=−.1; *P*<.001), evening snack (β=−.1; *P*<.001), and exercise (β=−.03; *P*<.001) exhibited significant weight loss over time. We found no significant differences in the body weight changes between users who used the free and paid versions of the app (β=−.2; *P*=.19).

**Conclusions:**

This study found that using the app has a significant effect on weight loss regardless of users’ country of residence. The results of this study suggest that the frequency of monitoring health-related behaviors by entering data into the app plays a pivotal role in losing weight. In conclusion, regardless of where users live and what versions of the app they use, it is important to monitor health-related behaviors by frequently entering data into the app to efficiently lose weight.

## Introduction

### Background

Annually, approximately 41 million people (or 71% of all deaths globally) die of noncommunicable diseases (NCDs), such as cardiovascular diseases, cancers, and diabetes [1]. Accordingly, poor diet quality and physical inactivity, which are the key factors for the prevalence of NCDs and mortality worldwide, are considered priority areas for global action [2]. Improving physical activity (PA) and dietary intake have been emphasized to prevent NCDs [3].

Given the global scale of NCDs, effective preventative interventions that can reach a wide range of populations at low costs are urgently needed [4]. It has been recognized that technology can support health improvements worldwide [1]. Specifically, because of the lowering prices of smartphones and the easy access they provide, mobile technology is expected to play a particularly important role in improving health-related behaviors in low-, middle-, and high-income countries [5]. Currently, smartphones are arguably the most prosperous and expeditiously adopted modern technology in the world. In low-income countries, access to smartphones increased from 4% to 94% from 2000 to 2015 [6].

### Objectives

The growth in mobile technologies has stimulated the growth of smartphone health and fitness apps [7]. The apps that target health promotion have become a central part of people’s lives and have demonstrated large increases in use [8]. According to previous studies, smartphone health apps have a positive impact on the improvement of health-related behaviors and outcomes, such as dietary intake, PA, and weight loss [9].

However, despite the growing use of apps that target health-related behavior change, the long-term effects of such apps on targeted health behaviors and outcomes, such as diet, PA, and weight loss, particularly among users in low-income countries, remains unclear [10,11]. Most previous studies were conducted in wealthy countries with highly developed technologies. To fill this gap in the literature, this study was conducted to compare the characteristics of adherence with using a smartphone app (Noom) and the effectiveness of the apps between low-income and Organization for Economic Co-operation and Development (OECD) countries.

The hypotheses tested in this study were as follows:

- H1: There would be differences in adherence to the use of self-monitoring data entry of diet, PA, and body weight between users from low-income countries and OECD countries.
- H2: There would be differences in body weight changes over time between users from low-income and OECD countries.
- H3: There would be differences in body weight changes between users of the paid and free versions of the app.

## Methods

### Study Design

This study was a retrospective cohort study that aimed to compare the use and effectiveness of a smartphone health app on changes in body weight over time between low-income and OECD countries.

### Setting

#### Low-Income Countries

The list of low-income countries was derived from a report by the International Statistical Institute. The gross national income, derived from the World Bank County classification, was used as a measure of a country’s income. Countries with gross national income per capita slightly over US $12,476 were considered as low-income countries [12]. In this study, we focused on data from 31 low-income countries (Multimedia Appendix 1).

#### OECD Countries

The OECD is an intergovernmental economic organization with 36 member countries [13]. Most OECD countries are high-income economies with a high human development index and are regarded as high-income countries [14]. The data of users from 32 OECD countries were included in this study (Multimedia Appendix 2).

### Participants

The data were provided by the Noom Coach (Noom Inc) app company. Individuals who used the Noom Coach app for 12 months between October 2012 and April 2014 and provided relevant data (demographic characteristics, exercise, dietary intake, and weight) were included. The participants used the exercise data entry function, dietary data entry function, or weight data entry function. As the age of 42 years is the default value in the age tab of the app, we excluded all users who indicated 42 years as their age, assuming that all these users did not correctly indicate their age when they started using the app. Accordingly, from 48,095 cases in the original data set, 4026 cases were removed. From the remaining 44,069 users, we selected 12,173 users who used the app for 12 months. On deletion of users who did not enter all necessary data (n=3638), the final data set contained a total of 8353 users.

### Intervention: Noom Coach

Noom Coach is a smartphone app for weight loss that tracks dietary intake, PA, and body weight. Created in 2012, this app is now available in 5 languages (English, Korean, Japanese, German, and Spanish) from the Google Play store; the iOS version is also available. With more than 10 million downloads worldwide, this app has been consistently ranked as the most effective weight loss app [15,16].

When app users log in for the first time, they are asked to enter the expected body weight and record their present body weight and height. During the period of using the Noom Coach app, users are requested to record their daily dietary intake and the number of footsteps as their PA. On the basis of the data entered by users, the app reports the trends in body weight changes, calories, and nutritional summaries. To support the achievement of the desired body weight, the app provides tailored feedback, including types of exercise. The app is available in 2 versions: free and paid. The free version includes functions such as food logging, weight tracking, and helpful tips. The paid version offers more services, including supportive advice from a specialist, new recipes of dietary intake, workout guides, tracking progress, and one-to-one coaching.

### Statistical Methods

All statistical analyses were performed using SPSS (version 24.0; IBM Corporation). Appropriate descriptive statistic analyses were conducted on baseline variables, such as age, sex, body weight, BMI, and the frequency of data entry on each section of diet and exercise. To analyze the differences between users of the app in OECD countries and low-income countries, independent two-tailed sample *t* tests were conducted. Moreover, the progression of body weight over time was described graphically. A linear mixed model (LMM) was used to evaluate the differences in body weight changes over time between the low-income and OECD countries. Statistical significance was determined at *P*<.05 (2-sided). Before conducting the analysis, all assumptions were checked and met. The dispersion of the outcome variable of body weight was checked before conducting the LMM. All assumptions were met to conduct an LMM.

LMM with random intercepts was used to evaluate the effects of time and the effects of gender, age, group (OECD countries vs low-income countries), frequency of exercise data entry, frequency of breakfast data entry, frequency of morning snack data entry, frequency of lunch data entry, frequency of afternoon snack data entry, frequency of dinner data entry, and frequency of evening snack data entry on the weight changes over time. From the unconditional model, the value of intraclass correlations was 0.9, which confirmed the use of the LMM for further analysis. From the results of the conditional model, the random effects model was selected for the LMM. Individual univariate LMMs were conducted with each independent variable to select all significant variables. All significant variables were entered into the LMM model, and then backward eliminations were conducted until the minimum values of Akaike information criterion and Bayesian information criterion were reached, indicating the best model that predicted the body weight changes over time.

### Variables

In this study, adherence to app use was defined as the frequency of exercise, diet (breakfast, breakfast snacks, lunch, afternoon snacks, dinner, and dinner snacks), or the frequency of weight data entry [11]. The outcome variable was body weight (kg) changes from baseline to 12 months. If there were no body weight indications at baseline or at 3, 6, 9, and 12 months, the values were replaced with the average weight calculated by averaging the body weight before and after 7 days at each time point. Age and sex were used as the self-reported baseline values. BMI (kg/m^2^) was calculated using weight in kilograms divided by height in meters squared. BMI was divided into the following 4 categories: underweight (≤18.5 kg), normal (18.5-24.9 kg), overweight (25-29.9 kg), and obese (≥30 kg) [17]. The frequencies of data entry for exercise, breakfast, morning snack, lunch, afternoon snack, dinner, and evening snack were the sum of the number of days with values every 3 months. The average calories for exercise, breakfast, morning snack, lunch, afternoon snack, dinner, and evening snack were calculated by averaging the calories consumed before and after 7 days at each time point.

## Results

### Characteristics of Participants

The baseline characteristics of the participants (N=8343) are summarized in Table 1. Of the 8343 users, 8041 (96.38%) were from OECD countries and 312 (3.88%) were from low-income countries. The mean ages of users from low-income countries and OECD countries were 32.43 years (SD 9.5; range 18-66) and 36.1 years (SD 11.7; range 13-76), respectively. Most users in each group were female (6024/8343, 72.2%). The mean values of body weight of users from low-income and OECD countries were 74.6 kg and 82.2 kg at baseline, respectively. Most users in low-income countries (208/312, 66.7%) and OECD countries (5468/8041, 68%) were obese.

**Table 1:** Demographic characteristics at baseline (N=8353).

Characteristics			Organization for Economic Co-operation and Development countries (n=8041)		Low-income countries (n=312)
Age (years), mean (SD; range)			36.1 (11.7; 13.0-76.0)		32.4 (9.5; 18.0-66.0)
**Gender, n (%)**					
	Male	2017 (25.08)		112 (35.9)	
	Female	6024 (74.92)		200 (64.1)	
Weight (kg), mean (SD; range)			82.2 (21.2; 39.0-188.7)		74.6 (17.1; 49.0-158.8)
**BMI (kg/m^2^), n (%)**					
	Underweight	63 (0.78)		2 (0.64)	
	Normal weight	1400 (17.41)		60 (19.23)	
	Overweight	1110 (13.80)		42 (13.46)	
	Obese	5468 (68)		208 (66.67)	
Frequency of exercise entry, mean (SD; range)			123.6 (97.4; 0.0-360.0)		126.2 (96.6; 0.0-360.0)
Frequency of breakfast data entry, mean (SD; range)			198.5 (99.6; 0.0-360.0)		199.3 (103.8; 0.0-360.0)
Frequency of morning snack data entry, mean (SD; range)			89.0 (85.8; 0.0-360.0)		114.8 (99.7; 0.0-360.0)
Frequency of lunch data entry, mean (SD; range)			183.1 (99.0; 0.0-360.0)		179.7 (104.5; 0.0-360.0)
Frequency of afternoon snack data entry, mean (SD; range)			102.0 (84.9; 0.0-360.0)		120.4 (97.1; 0.0-360.0)
Frequency of dinner data entry, mean (SD; range)			152.5 (100.8; 0.0-360.0)		149.4 (106.3; 0.0-360.0)
Frequency of evening snack data entry, mean (SD; range)			57.5 (70.1; 0.0-360.0)		62.7 (76.0; 0.0-360.0)
Exercise, mean (SD; range)			305.8 (192.2; 0.0-1300.0)		280.4 (190.1; 0.0-1300.0)
Breakfast, mean (SD; range)			288.1 (110.3; 0.0-700.0)		293.8 (112.0; 0.0-700.0)
Morning snack, mean (SD; range)			179.7 (95.4; 0.0-1244.4)		187.4 (107.6; 0.0-1200.0)
Lunch, mean (SD; range)			413.9 (151.0; 0.0-1419.7)		226.6 (107.2; 0.0-1400.0)
Afternoon snack, mean (SD; range)			225.0 (122.0; 0.0-1500.0)		401.9 (171.2; 0.0-1108.2)
Dinner, mean (SD; range)			458.8 (173.0; 0.0-1436.7)		223.7 (125.0; 0.0-714.7)
Evening snack, mean (SD; range)			235.5 (123.7; 0.0-978.0)		157.9 (74.6; 0.0-157.9)

The average frequencies of data entry of users from OECD countries for exercise, breakfast, morning snack, lunch, afternoon snack, dinner, and evening snack were 123.6, 198.5, 89.0, 183.1, 102.0, 152.5, and 57.5, respectively. The average frequencies of data entry of users from low-income countries for exercise, breakfast, morning snack, lunch, afternoon snack, dinner, and evening snack were 126.2, 199.3, 114.8, 179.7, 120.4, 149.4, and 62.7, respectively. The mean calories of exercise, breakfast, morning snack, lunch, afternoon snack, dinner, and evening snack of users from OECD countries were 305.8, 288.1, 179.7, 413.9, 225.0, 458.8, and 235.5, respectively. The mean calories of exercise, breakfast, morning snack, lunch, afternoon snack, dinner, and evening snack of users from low-income countries were 280.4, 293.8, 187.4, 226.6, 401.9, 223.7, and 157.9, respectively. The values corresponding to each item represent the average value over a period when users use the app.

### Comparison of Frequency of Self-Monitoring Data Entry Between Low-Income and OECD Countries at Each Time Point

Independent two-tailed sample *t* tests were conducted to compare the frequency of data entry and calories between users from low-income and OECD countries. There were significant differences in the frequency of data entering for breakfast (*t*_8351_=−2.6; *P*=.009), morning snack (*t*_8351_=3.1; *P*=.002), lunch (*t*_8351_=−3.1; *P*=.002), and dinner (*t*_8351_=−2.9; *P*=.003) data between baseline and 3 months. At this time point, users from OECD countries entered their data (except for those on morning snack) more frequently than those from low-income countries. Between 3 and 6 months, users from low-income countries entered morning snack (*t*_8351_=5.0; *P*<.001) and afternoon snack data (*t*_8351_=3.6; *P*<.001) significantly more frequently. Users in low-income countries entered morning snack (*t*_8351_=4.3; *P*<.001) and afternoon snack data (*t*_8351_=2.8; *P*=.005) more times than those in OECD countries between 6 and 9 months. Between 9 and 12 months, there was a significant difference in the values for morning snack (*t*_8351_=3.8; *P*<.001) and afternoon snack data (*t*_8351_=2.3; *P*<.02) entering between the users from low-income and OECD countries (Table 2). These results support hypothesis 1, as there were differences in adherence to the use of self-monitoring data entry between users from low-income countries and OECD countries.

**Table 2:** Results of independent two-tailed *t* tests to compare the difference in frequency of data entry between Organization for Economic Co-operation and Development (n=8041) and low-income countries (n=312).

Characteristics	Time 1 (baseline to 3 months), mean (SD)			Time 2 (3-6 months), mean (SD)			Time 3 (6-9 months), mean (SD)			Time 4 (9-12 months), mean (SD)	
	OECD^a^	Low-income	OECD		Low-income	OECD		Low-income	OECD		Low-income
Frequency of exercise data entry	35.1 (23.0)	36.2 (24.5)	28.4 (23.6)		29.7 (24.3)	22.2 (22.5)		23.3 (23.0)	18.5 (21.1)		19.4 (21.1)
Frequency of breakfast data entry	51.5 (30.0)^b^	46.9 (32.1)^b^	50.9 (28.1)		51.7 (27.3)	40.0 (29.7)		40.4 (30.5)	32.9 (29.1)		33.9 (29.9)
Frequency of morning snack data entry	22.4 (22.2)^b^	26.4 (26.4)^b^	21.7 (22.3)^b^		28.2 (25.8)^b^	16.4 (20.5)^b^		21.5 (24.6)^b^	13.4 (19.2)^b^		17.8 (23.4)^b^
Frequency of lunch data entry	48.4 (29.4)^b^	43.1 (32.1)^b^	47.1 (47.5)		47.5 (28.4)	36.2 (28.9)		36.8 (30.3)	29.5 (28.2)		30.3 (29.3)
Frequency of afternoon snack data entry	27.7 (23.0)	29.2 (26.4)	25.5 (22.6)^b^		30.3 (24.9)^b^	18.8 (21.2)^b^		22.3 (24.3)^b^	15.2 (19.8)^b^		17.9 (22.6)^b^
Frequency of dinner data entry	42.8 (29.1)^b^	37.8 (30.7)^b^	38.7 (28.0)		38.8 (22.6)	29.1 (27.8)		29.3 (28.2)	23.6 (26.3)		23.8 (27.1)
Frequency of evening snack data entry	16.5 (18.1)	17.1 (20.5)	13.6 (17.5)		15.0 (19.3)	21.1 (16.8)		21.1 (18.0)	8.0 (14.9)		8.9 (16.1)

^a^OECD: Organization for Economic Co-operation and Development.

^b^Significant at the *P*<.05 level.

### Body Weight Changes Between Users in Low-Income and OECD Countries From Baseline to 12 Months

**Figure 1:**
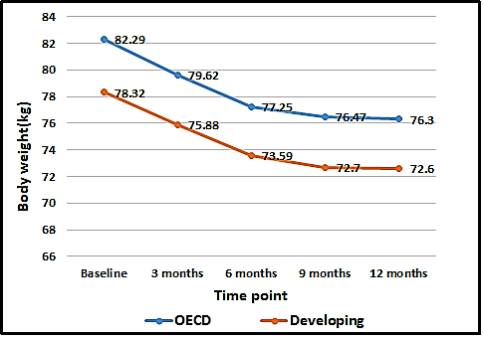
Comparison of body weight changes over time between Organization for Economic Co-operation and Development (n=8041) and low-income countries (n=312). OECD: Organization for Economic Co-operation and Development.

Figure 1 shows the body weight (kg) changes of users from the low-income and OECD countries at each time point. The body weight of users from the low-income and OECD countries was 78.3 and 82.3 at baseline, respectively. From baseline to 6 months, users in both types of countries exhibited a dramatic reduction in their body weight, about 5 kg, which then slightly decreased until 12 months (Figure 1). There were no statistically significant differences in the degree of weight loss between users from the low-income and OECD countries at each time point (baseline to 3 months, 3-6 months, 6-9 months, and 9-12 months; Table 3).

**Table 3:** Difference in the degree of weight loss between Organization for Economic Co-operation and Development (n=8041) and low-income (n=312) countries at each time point.

Time	Organization for Economic Co-operation and Development countries, mean (SD)	Low-income countries, mean (SD)	*t* test (*df*)	*P* value	95% CI
Baseline to 3 months	−2.7 (5.1)	−2.4 (7.7)	0.7 (8351)	.46	−0.4 to 0.8
3-6 months	−2.4 (3.8)	−2.3 (3.2)	0.4 (8351)	.67	−0.3 to 0.5
6-9 months	−0.8 (3.7)	−0.9 (2.1)	−0.5 (8351)	.63	−0.5 to 0.3
9-12 months	−0.2 (4.8)	−0.03 (2.1)	0.6 (8351)	.54	−0.4 to 0.7

### Comparison of Body Weight Changes Over Time Between Users From Low-Income and OECD Countries and Between Users of the Paid and Free Versions of the App

The change in body weight over time was not significantly different between the users from low-income and OECD countries (β=−.2; *P*=.19). However, there were differences in body weight changes over time according to adherence to the app. For every increase of 1 unit in frequency of exercise (β=−.004; *P*<.001), lunch (β=−.01; *P*<.001), dinner (β=−.01; *P*<.001), evening snack (β=−.01; *P*<.001), or weight (β=−.01; *P*<.001), body weight statistically significantly decreased. The changes in body weight differed by gender, as demonstrated by the interaction between time and sex (β=.7; *P*<.001). For every increase of 1 unit in age, the body weight increased by 0.002 (*P*=.03; Table 4). There was no significant difference in body weight changes over time based on the version of the app (free version vs paid version; β=−.01; *P*=.91). On the basis of the results, the hypothesis that there would be differences in body weight change over time according to smartphone adherence between users from low-income and OECD countries and the hypothesis that there would be differences in body weight changes between users of the paid and free versions of the app had to be rejected.

**Table 4:** Compare the difference in body weight changes over time between low-income (n=312) and Organization for Economic Co-operation and Development (n=8041) countries.

Parameter	Estimate (SE; 95% CI)	*t* test (*df*)	*P* value
Intercept	89.8 (1.2; 87.4–92.3)	72.2 (8380.0)	<.001
Time	−1.7 (0.1; −1.9 to −1.4)	−12.8 (8524.7)	<.001
Age	0.1 (0.01; 0.04–0.1)	6.6 (8347.3)	<.001
Gender	−19.0 (0.5; −20.0 to −18.0)	−37.4 (8809.2)	<.001
OECD^a^	5.7 (1.2; 3.4–8.0)	4.8 (8346.2)	<.001
Frequency of lunch data entry	−0.04 (0.01; −0.1 to −0.03)	−7.7 (30380.0)	<.001
Frequency of dinner data entry	0.03 (0.01; 0.02–0.1)	5.2 (30493.1)	<.001
Frequency of weight data entry	−0.02 (0.003; −0.02 to −0.01)	−5.0 (31933.7)	<.001
Frequency of evening snack data entry	0.02 (0.01; 0.01–0.03)	3.2 (31694.5)	.001
Interaction between time and gender	0.7 (0.1; 0.6–0.8)	12.7 (8369.0)	<.001
Interaction between time and frequency of lunch data entry	0.01 (0.001; 0.01–0.01)	5.7 (32911.1)	<.001
Interaction between time and frequency of dinner data entry	−0.01 (0.002; −0.01 to −0.01)	−4.9 (33057.6)	<.001
Interaction between time and frequency of body weight data entry	−0.01 (0.001; −0.01 to −0.003)	−4.1 (33075.5)	<.001
Interaction between time and frequency of evening snack data entry	−0.01 (0.002; −0.01 to −0.004)	−4.4 (33007.3)	<.001
Interaction between time and frequency of exercise data entry	−0.003 (0.0004; −0.004 to −0.003)	−7.6 (33510.8)	<.001
Interaction between time and age	0.002 (0.001; −0.0001 to 0.003)	1.8 (8299.9)	.03
Interaction between time and OECD	−0.2 (0.12; −0.4 to 0.1)	−1.3 (8304.4)	.19

^a^OECD: Organization for Economic Co-operation and Development.

### Comparison of Consumed Calories Between Users From the Low-Income and OECD Countries at Each Time Point

There were significant differences in calorie consumption for each category between the users from low-income and OECD countries. Between baseline and 3 months, users from OECD countries consumed significantly more calories for breakfast (*t*_8351_=−2.5; *P*=.01), lunch (*t*_8351_=−3.5; *P*<.001), afternoon snack (*t*_8351_=−2.0; *P*=.04), dinner (*t*_8351_=−6.7; *P*<.001), and evening snacks (*t*_8351_=−2.2; *P*=.03). Compared with users from low-income countries, users from OECD countries also consumed significantly more calories for exercise (*t*_8351_=−2.7; *P*=.008) and dinner (*t*_8351_=−5.2; *P*<.001) between 3 and 6 months. There was also a statistically significant difference in calories of exercise (*t*_8351_=−2.6; *P*=.01) and dinner (*t*_8351_=−3.9; *P*<.001) for both groups between 6 and 9 months. Users from OECD countries consumed more calories for exercise and dinner. Between 9 months and 12 months, users from the OECD countries consumed significantly higher amounts of dinner calories than users from low-income countries (*t*_8351_=−4.7; *P*<.001; Table 5).

**Table 5:** Results of independent two-tailed *t* tests of average consumed calories between Organization for Economic Co-operation and Development (n=8041) and low-income countries (n=312).

Characteristics	Time 2 (baseline to 3 months), mean (SD)		Time 3 (3-6 months), mean (SD)		Time 4 (6-9 months), mean (SD)		Time 5 (9-12 months), mean (SD)	
	OECD^a^	Low-Income	OECD	Low-Income	OECD	Low-Income	OECD	Low-Income
Calories of exercise	277.0 (189.1)	259.8 (192.6)	286.3 (217.9)^b^	252.9 (182.8)^b^	277.6 (232.3)^b^	243.4 (202.7)^b^	270.4 (241.1)	231.6 (214.9)
Calories of breakfast data	263.6 (130.8)^b^	245.0 (145.1)^b^	280.8 (125.7)	280.0 (126.7)	276.5 (134.2)	280.3 (141.1)	270.1 (142.3)	281.8 (151.9)
Calories of morning snack	152.1 (149.6)	149.6 (122.1)	169.1 (120.8)	169.4 (105.0)	158.5 (126.6)	171.6 (132.7)	149.6 (136.4)	162.4 (132.5)
Calories of lunch	366.8 (175.9)^b^	331.0 (192.1)^b^	402.0 (163.8)	388.0 (170.4)	392.4 (382.6)	382.6 (199.1)	381.5 (197.9)	374.6 (210.2)
Calories of afternoon snack	188.9 (113.7)^b^	175.2 (121.6)^b^	207.4 (118.4)	216.2 (108.8)	197.8 (132.6)	203.8 (133.6)	185.6 (140.0)	188.5 (134.8)
Calories of dinner	402.5 (208.4)^b^	321.4 (192.9)^b^	442.2 (197.2)^b^	383.1 (176.6)^b^	417.7 (223.0)^b^	367.5 (227.0)^b^	405.4 (244.1)^b^	339.1 (235.8)^b^
Calories of evening snack	192.4 (138.7)^b^	174.3 (146.2)^b^	203.0 (149.4)	187.9 (152.3)	178.2 (171.4)	173.8 (163.0)	162.3 (166.3)	149.5 (164.8)

^a^OECD: Organization for Economic Co-operation and Development.

^b^Significant at the *P*<.05 level.

### Comparison of the Total Frequency of Data Entry and Overall Calorie Consumption Between Users From Low-Income and OECD Countries

Independent two-tailed sample *t* tests were conducted to compare the total frequency and overall calorie consumption between users from low-income and OECD countries. There was a statistically significant difference in the frequency of data entry of morning snack (*t*_8351_=−5.4; *P*<.001) and afternoon snack (*t*_8351_=3.9; *P*<.001) between the two groups of users (Table 6). There were also significant differences in the average calories for exercise (*t*_8351_=−2.4; *P*=.02) and dinner (*t*_8351_=−5.9; *P*<.001; Table 7).

**Table 6:** Results of independent two-tailed *t* tests to compare the total frequency of data entry between Organization for Economic Co-operation and Development (n=8041) and low-income countries (n=312).

Characteristics	Organization for Economic Co-operation and Development countries (n=8041), mean (SD)	Low-income countries (n=321), mean (SD)	*t* test (*df*)	*P* value	*t*-value (95% CI)
Total frequency of exercise data entry	123.6 (97.4)	126.2 (96.6)	0.1 (8351)	.65	−8.4 to 13.6
Total frequency of breakfast data entry	198.5 (99.4)	199.3 (103.8)	−0.2 (8351)	.88	−10.4 to 12.1
Total frequency of morning snack data entry	88.0 (85.1)	114.8 (99.7)	−5.4 (8351)	<.001	17.1 to 36.5
Total frequency of lunch data entry	183.2 (98.7)	179.7 (104.5)	−0.9 (8351)	.53	−14.8 to 7.6
Total frequency of afternoon snack data entry	101.3 (84.4)	120.4 (97.1)	3.9 (8351)	<.001	9.5 to 28.7
Total frequency of dinner data entry	152.6 (100.6)	149.36 (106.3)	1.1 (8351)	.58	−15.3 to 8.8
Total frequency of evening snack data entry	57.3 (69.9)	62.74 (76.0)	−0.3 (8351)	.18	−7.3 to 9.2

**Table 7:** Results of independent two-tailed *t* tests to compare the overall calories between Organization for Economic Co-operation and Development (n=8041) and low-income countries (n=312).

Characteristics	Organization for Economic Co-operation and Development countries, mean (SD)	Low-income countries, mean (SD)	*t* test (*df*)	*P* value	*t*-value (95% CI)
Exercise	306.8 (192.2)	280.4 (190.1)	−2.4 (8351)	.02	−48.2 to −4.7
Breakfast	287.9 (110.2)	293.8 (112.0)	1.0 (8351)	.35	−6.6 to 18.4
Morning snack	179.4 (94.8)	187.4 (107.6)	1.1 (8351)	.15	−2.8 to 18.8
Lunch	414.1 (150.5)	408.6 (163.4)	−0.6 (8351)	.53	−22.5 to 11.6
Afternoon snack	224.9 (122.5)	226.6 (107.2)	1.2 (8351)	.81	−12.1 to 15.5
Dinner	461.0 (172.7)	401.9 (171.2)	−5.9 (8351)	<.001	−78.7 to −39.6
Evening snack	236.0 (123.6)	223.7 (125.0)	−1.8 (8351)	.09	−26.2 to 1.8

## Discussion

### Principal Findings

The results of this study suggest that there are significant differences in using the app between users from low-income and OECD countries. At most time points, users from low-income countries entered more data into the app than users from OECD countries. However, all users exhibited a decrease in the frequency of app use over time. There has been insufficient research on adherence to mobile health (mHealth) apps, especially regarding users in low-income countries. Accordingly, it is difficult to understand the level of adherence to the use of mHealth apps and to compare differences in adherence rates among different groups of users. However, achieving long-term health-related goals, such as weight loss, requires constant and committed participation in using the mHealth app. In addition, the low retention rate of using mHealth apps has been recognized as a critical problem. Therefore, further research on adherence to using mHealth apps is needed.

According to a recent study, more than two-thirds of people who downloaded an mHealth app used it only once or stopped using it within a short time [18]. Similarly, Lee et al [19] reported the use of mHealth apps to gradually reduce over time. To benefit from an mHealth app, users should continue to use it for a sufficient amount of time so that it can be incorporated into their daily lives [20]. Furthermore, users should put much effort into using mHealth apps for a long time because changing habitual behavior takes a substantial amount of time [19]. Accordingly, researchers and app developers should investigate and adopt essential features that would encourage users to keep using mHealth apps to accomplish their health outcomes.

In our results, the frequency of data entry was significantly associated with weight loss over time. Specifically, users who frequently monitored their lunch, dinner, body weight, evening snack, or exercise exhibited significant weight loss over time. As previous studies established that eating a late and large amount of dinner is associated with weight gain [21,22], it can be assumed that those users who more frequently track their exercise and dinner calories try to increase their movement and reduce dinner calories or make an effort to eat dinner in small quantities and earlier than usual. Similar to our findings, a systematic review found a significant relationship between the frequency of self-monitoring for diet and PA and weight loss [23]. Furthermore, Conroy et al [24] found that higher mean rates of PA self-monitoring were associated with a greater reduction in weight. Moreover, Peterson et al [25] reported that the total number of food records can be a predictor of weight loss, regardless of the type of meals. On the basis of this evidence, it can be concluded that people who more consistently track their food, exercise, and body weight by using the app are more likely to lose weight [26].

In fact, there is insufficient research on the importance of having separate data entries for meals and snacks on weight loss. However, it can be assumed that people can understand their current eating habits and the nutrient value of food by tracking meals and snacks. Through this process, they might enhance their ability to balance total calories and macronutrients throughout the day and reduce their body weight in the long term [27]. Regular weighing is another essential and simple way to lose weight by boosting motivation [28]. A study found that people who never weighed themselves or only weighed themselves once per week did not exhibit weight reduction. However, those who weighed themselves almost daily exhibited significant weight loss within 1 year [29]. The monitoring and logging of exercise can encourage people to move more throughout the day; therefore, people can efficiently reduce their body weight [30]. To summarize, many studies have found that monitoring health-related behaviors or weight can increase people’s perceptions of the effects of changing behaviors related to weight loss [29,31,32].

It was found that there were no significant differences in weight change between users living in low-income or OECD countries. Specifically, we found that self-monitoring of PA, diet, or body weight through a smartphone app can be a useful tool to lose weight regardless of the users’ country of residence. Users from the low-income and OECD countries showed statistically significant weight loss at 9 months after starting using the app; however, users from OECD countries regained their weight and returned to their original body weight in 12 months. Lifestyle, including not only diet and PA patterns, varies considerably across countries [33]. Specifically, although high-income countries generally have better diets based on healthy foods, they also have substantially poorer diets because of a higher intake of unhealthy foods compared with low-income countries [34]. In addition, high-income countries are often linked to low levels of PA levels [35]. These reasons could have influenced the weight gain of users from OECD countries in this study. However, further research on the reasons that made users from OECD countries gain weight is needed.

In this study, we assumed that the users of the paid version of the app would show more body weight reduction than users who used the free version of the app. Our prediction was based on the fact that the paid version offers a variety of features, including customized diet planning, one-on-one coaching, social support, weight recording logs, and food and exercise tracking, whereas the free version only allows users to log and track their data on diet, exercise, and weight [36]. However, the results showed that there were no statistically significant differences in body weight changes over time between the users of the 2 versions of the app. A previous systematic review found that self-monitoring of diet, exercise, and weight is the core feature of behavioral weight loss intervention programs [37]. However, there is a lack of information about the features of smartphone health apps that are the most valuable in terms of weight loss. To develop an efficient and effective app for weight loss, further research is required to identify such features.

The imminent global threat of NCDs calls for urgent solutions that would extend existing health systems into the community [38]. In recent years, smartphone health care apps have attracted significant attention as effective interventions to prevent NCDs [39]. However, despite the widespread use of smartphone apps as interventions to lose weight, previous research on body weight changes according to using smartphone health apps in low-income countries remains scarce [40]. This study compared the use of an app and its effectiveness on body weight changes over time in low-income and OECD countries. The results showed no significant differences in the use and effectiveness of the app between users from the two groups of countries. Although the app provides a specialized diet and PA monitoring programs in many languages and targets diverse populations around the world, the number of users in OECD countries is more than 4 times the number in low-income countries. Although it is difficult to conclude that our data set contained all users’ information about the app, this information provides a need to consider the barriers and challenges of smartphone use in low-income countries. As smartphone apps can provide extraordinary health opportunities to users from low-income countries, which seriously lack health infrastructure and clinical resources, many efforts to enhance the use of smartphone health apps are needed so that these apps to reach their full potential in low-income countries [6].

This study has several limitations. First, as our study was a retrospective cohort study, the results may be limited by the observational nature of the data set. To accurately evaluate the effectiveness of the app, well-developed randomized controlled trials should be conducted. Second, our data set included only a representative selection of OECD and low-income countries, which might have affected the generalizability of our findings. Third, our analysis did not include some important demographic factors, socioeconomic factors, and lifestyle variables that could affect the use of the smartphone app and body weight changes. Fourth, the users downloaded and used the app with the intention of monitoring their diet and PA and to voluntarily lose weight. Therefore, this might have made the results significant in this analysis. Fifth, it is possible that users may engage in a suitable diet and PA without logging or self-reporting. Finally, the analyzed data were self-reported, which might have affected the accuracy of our conclusions. Despite the aforementioned limitations, the results of this study provide valuable insights for investigators, engineers, politicians, policymakers, government authorities, health care providers, and the general population worldwide in terms of highlighting the benefits of using health care smartphone apps in health promotion. Our results also highlight the strong potential of the studied app [41].

### Conclusions

In conclusion, this study is the first to investigate the effectiveness of using a smartphone app on losing body weight between users from low-income and OECD countries. On the basis of the results, it can be concluded that the frequent input of self-monitoring data, the main function of the Noom app, can be an effective approach to weight loss. We also found no significant differences in body weight changes between users from the two groups of countries, suggesting that the smartphone app can be an effective and general way to lose weight regardless of the users’ country of residence. In addition, we also found that using even the free version of the app increases the frequency of self-monitoring and thus positively affects weight loss.

## Appendix

Multimedia Appendix 1 Low-income countries.

Multimedia Appendix 2 Organization for Economic Co-operation and Development countries.

## Abbreviations

LMM: linear mixed model
mHealth: mobile health
NCD: noncommunicable disease
OECD: Organization for Economic Co-operation and Development
PA: physical activity

## References

[1] (2018). Noncommunicable Diseases. World Health Organization.

[2] Lim S, Vos T, Flaxman D, Danaei G, Shibuya K, Adair-Rohani H, Amann M, Anderson HR, Andrews KG, Aryee M, Atkinson C, Bacchus LJ, Bahalim AN, Balakrishnan K, Balmes J, Barker-Collo S, Baxter A, Bell ML, Blore JD, Blyth F, Bonner C, Borges G, Bourne R, Boussinesq M, Brauer M, Brooks P, Bruce MG, Brunekreef B, Bryan-Hancock C, Bucello C, Buchbinder R, Bull F, Burnett RT, Byers TE, Calabria B, Carapetis J, Carnahan E, Chafe Z, Charlson F, Chen H, Chen JS, Cheng AT, Child JC, Cohen A, Colson KE (2012). A comparative risk assessment of burden of disease and injury attributable to 67 risk factors and risk factor clusters in 21 regions, 1990-2010: a systematic analysis for the Global Burden of Disease Study 2010. Lancet.

[3] Lachat C, Otchere S, Roberfroid D, Abdulai A, Seret FM, Milesevic J, Xuereb G, Candeias V, Kolsteren P (2013). Diet and physical activity for the prevention of noncommunicable diseases in low- and middle-income countries: a systematic policy review. PLoS Med.

[4] Morrison LG, Hargood C, Lin SX, Dennison L, Joseph J, Hughes S, Michaelides DT, Johnston D, Johnston M, Michie S, Little P, Smith PW, Weal MJ, Yardley L (2014). Understanding usage of a hybrid website and smartphone app for weight management: a mixed-methods study. J Med Internet Res.

[5] Mayes J, White A (2017). How smartphone technology is changing health care in developing countries. Journal of Glob Heal.

[6] Kahn J, Yang J (2010). Mobile health needs and opportunities in developing countries. Health Aff.

[7] Gruessner V (2015). The Advantages of Mobile Health Apps Today and Tomorrow. mhealth Intelligence.

[8] Kaplan W (2006). Can the ubiquitous power of mobile phones be used to improve health outcomes in developing countries?. Global Health.

[9] Schoeppe S, Alley S, Van Lippevelde W, Bray NA, Williams SL, Duncan MJ, Vandelanotte C (2016). Efficacy of interventions that use apps to improve diet, physical activity and sedentary behaviour: a systematic review. Int J Behav Nutr Phys Act.

[10] Milne-Ives M, Lam C, de Cock C, van Velthoven MH, Meinert E (2020). Mobile apps for health behavior change in physical activity, diet, drug and alcohol use, and mental health: systematic review. JMIR Mhealth Uhealth.

[11] Ustulin M, Keum C, Woo J, Woo J, Rhee SY (2017). Effects of climatic variables on weight loss: a global analysis. Sci Rep.

[12] Bhurosy T, Jeewon R (2014). Overweight and obesity epidemic in developing countries: a problem with diet, physical activity, or socioeconomic status?. Sci World J.

[13] (2018). List of OECD Member Countries-ratification of the Convention on the OECD. The Organisation for Economic Co-operation and Development.

[14] (2018). World Economic Outlook Database. International Monetary Fund.

[15] Pagoto S, Schneider K, Jojic M, DeBiasse M, Mann D (2013). Evidence-based strategies in weight-loss mobile apps. Am J Prev Med.

[16] Chin SO, Keum C, Woo J, Park J, Choi HJ, Woo J, Rhee SY (2016). Successful weight reduction and maintenance by using a smartphone application in those with overweight and obesity. Sci Rep.

[17] BMI Formula. Centers for Disease Control and Prevention.

[18] Byrnes N (2014). Mobile Health's Growing Pains. MIT Technol Review.

[19] Lee K, Kwon H, Lee B, Lee G, Lee JH, Park YR, Shin S (2018). Effect of self-monitoring on long-term patient engagement with mobile health applications. PLoS One.

[20] Prochaska JO, DiClemente CC, Norcross JC (1992). In search of how people change: applications to addictive behaviors. Am Psychol.

[21] Jakubowicz D, Barnea M, Wainstein J, Froy O (2013). High caloric intake at breakfast vs dinner differentially influences weight loss of overweight and obese women. Obesity (Silver Spring).

[22] (2020). Eating an Early Dinner Can Help You Burn Fat, Lower Your Blood Sugar. Healthline.

[23] Burke LE, Wang J, Sevick MA (2011). Self-monitoring in weight loss: a systematic review of the literature. J Am Diet Assoc.

[24] Conroy M, Yang K, Burke L (2011). Physical activity self-monitoring and weight loss: 6-months results of the SMART trial. Med Sci Sport Exerc.

[25] Peterson ND, Middleton KR, Nackers LM, Medina KE, Milsom VA, Perri MG (2014). Dietary self-monitoring and long-term success with weight management. Obesity (Silver Spring).

[26] Wharton CM, Johnston CS, Cunningham BK, Sterner D (2014). Dietary self-monitoring, but not dietary quality, improves with use of smartphone app technology in an 8-week weight loss trial. J Nutr Educ Behav.

[27] Lesko A (2019). 8 Reasons Why You Should Try Tracking Your Food. Fit-Flavors.

[28] Jenny (2017). What to Track When You're Losing Weight. Progress Blog.

[29] American HA (2018). Daily Weighing May Be Key to Losing Weight. Sicence News.

[30] Hartmann-Boyce J, Boylan A, Jebb SA, Aveyard P (2019). Experiences of self-monitoring in self-directed weight loss and weight loss maintenance: systematic review of qualitative studies. Qual Health Res.

[31] Pagoto S, Schneider K, Jojic M, DeBiasse M, Mann D (2013). Evidence-based strategies in weight-loss mobile apps. Am J Prev Med.

[32] Aromatario O, Van Hoye A, Vuillemin A, Foucaut A, Crozet C, Pommier J, Cambon L (2019). How do mobile health applications support behaviour changes? A scoping review of mobile health applications relating to physical activity and eating behaviours. Public Health.

[33] Maher A, Sridhar D (2012). Political priority in the global fight against non-communicable diseases. J Glob Health.

[34] (2015). The Lancet Global Health: Unhealthy Eating Habits Outpacing Healthy Eating Patterns in Most World Regions. The Lancet.

[35] (2016). Physical Activity Fact Sheet. World Health Organization.

[36] What is Noom Diet Plan? A Nutritionist Honest Review. For Care Education and Research.

[37] Burke LE, Wang J, Sevick MA (2011). Self-monitoring in weight loss: a systematic review of the literature. J Am Diet Assoc.

[38] Kwan A (2017). Mhealth Opportunities for Non-communicable Diseases Among the Eldery. Click Medix.

[39] Dennison L, Morrison L, Conway G, Yardley L (2013). Opportunities and challenges for smartphone applications in supporting health behavior change: qualitative study. J Med Internet Res.

[40] Xiao Y, Zhao N, Wang H, Zhang J, He Q, Su D, Zhao M, Wang L, Zhang X, Gong W, Hu R, Yu M, Ding G, Cong L, Ye Z (2013). Association between socioeconomic status and obesity in a Chinese adult population. BMC Public Health.

[41] Latif S, Rana R, Qadir J, Ali A, Imran MA, Younis MS (2017). Mobile health in the developing world: review of literature and lessons from a case study. IEEE Access.

