# Supplementary material for: Effect of Adherence to Smartphone App Use on the Long-term Effectiveness of Weight Loss in Developing and OECD Countries: Retrospective Cohort Study
Source: JMIR Mhealth Uhealth. 2021 Jul 12;9(7):e13496. doi: 10.2196/13496 (PMC8314148; doi:10.2196/13496)
Supplement: Multimedia Appendix 1 [file mhealth_v9i7e13496_app1.docx]

**Appendix A**

**Developing Countries**

| **Frequency** | **Frequency** | **Percent** |
| --- | --- | --- |
| United Arab Emirates | 4 | .1 |
| Argentina | 2 | .1 |
| Bosnia and Herzegovina | 1 | .01 |
| Brazil | 6 | 3.2 |
| Chile | 4 | 2.2 |
| China | 1 | .5 |
| Colombia | 1 | .5 |
| Dominican Republic | 3 | 1.6 |
| Egypt | 2 | 1.1 |
| Estonia | 1 | .5 |
| Hongkong | 11 | .1 |
| Croatia | 17 | .2 |
| Indonesia | 4 | .1 |
| India | 37 | .5 |
| Iran | 1 | .1 |
| Iceland | 2 | .1 |
| Liechtenstein | 1 | .1 |
| North Macedonia | 1 | .1 |
| Malaysia | 4 | .1 |
| Philippines | 33 | .4 |
| Pakistan | 1 | .1 |
| Romania | 16 | .2 |
| Serbia | 6 | .1 |
| Russia | 51 | .6 |
| South Africa | 45 | .5 |
| Singapore | 4 | .1 |
| Thailand | 5 | .1 |
| Taiwan | 8 | .1 |
| Ukraine | 5 | .1 |
| Uruguay | 1 | .1 |
| Venezuela | 1 | .1 |
