# Supplementary material for: Effect of Adherence to Smartphone App Use on the Long-term Effectiveness of Weight Loss in Developing and OECD Countries: Retrospective Cohort Study
Source: JMIR Mhealth Uhealth. 2021 Jul 12;9(7):e13496. doi: 10.2196/13496 (PMC8314148; doi:10.2196/13496)
Supplement: Multimedia Appendix 2 [file mhealth_v9i7e13496_app2.docx]

**Appendix B**

OECD countries

| **Name** | **Frequency** | **Percent** |
| --- | --- | --- |
| Austria | 72 | .9 |
| Australia | 159 | 1.9 |
| Belgium | 15 | .2 |
| Canada | 172 | 2.1 |
| China | 63 | .8 |
| Czechia | 8 | .1 |
| Deutsch | 1591 | 19.0 |
| Denmark | 17 | .2 |
| Estonia | 6 | .1 |
| Spain | 91 | 1.1 |
| France | 68 | .8 |
| United Kingdom | 769 | 9.2 |
| Hungary | 17 | .2 |
| Ireland | 31 | .4 |
| Israel | 40 | .5 |
| Italy | 63 | .8 |
| Japan | 137 | 1.6 |
| Korea, republic of | 1221 | 14.6 |
| Lithuania | 11 | .1 |
| Luxembourg | 1 | .1 |
| Latvia | 8 | .1 |
| Mexico | 1 | .1 |
| Netherlands | 91 | 1.1 |
| Norway | 19 | .2 |
| New Zealand | 38 | .5 |
| Poland | 30 | .4 |
| Portugal | 14 | .2 |
| Sweden | 35 | .4 |
| Slovenia | 15 | .2 |
| Slovakia | 6 | .1 |
| Turkey | 13 | .2 |
| United States | 3164 | 37.9 |
